# Inhibition of Plasminogen Activator Inhibitor-1 Attenuates Transforming Growth Factor-β-Dependent Epithelial Mesenchymal Transition and Differentiation of Fibroblasts to Myofibroblasts

**DOI:** 10.1371/journal.pone.0148969

**Published:** 2016-02-09

**Authors:** Keitaro Omori, Noboru Hattori, Tadashi Senoo, Yusuke Takayama, Takeshi Masuda, Taku Nakashima, Hiroshi Iwamoto, Kazunori Fujitaka, Hironobu Hamada, Nobuoki Kohno

**Affiliations:** 1 Department of Molecular and Internal Medicine, Institute of Biomedical & Health Sciences, Hiroshima University, Hiroshima, Japan; 2 Department of Physical Analysis and Therapeutic Sciences, Graduate School of Biomedical & Health Sciences, Hiroshima University, Hiroshima, Japan; Helmholtz Zentrum München, GERMANY

## Abstract

Transforming growth factor-β (TGF-β) is central during the pathogenesis of pulmonary fibrosis, in which the plasminogen activator inhibitor-1 (PAI-1) also has an established role. TGF-β is also known to be the strongest inducer of PAI-1. To investigate the link between PAI-1 and TGF-β in fibrotic processes, we evaluated the effect of SK-216, a PAI-1-specific inhibitor, in TGF-β-dependent epithelial-mesenchymal transition (EMT) and fibroblast to myofibroblast differentiation. In human alveolar epithelial A549 cells, treatment with TGF-β induced EMT, whereas co-treatment with SK-216 attenuated the occurrence of EMT. The inhibition of TGF-β-induced EMT by SK-216 was also confirmed in the experiment using murine epithelial LA-4 cells. Blocking EMT by SK-216 inhibited TGF-β-induced endogenous production of PAI-1 and TGF-β in A549 cells as well. These effects of SK-216 were not likely mediated by suppressing either Smad or ERK pathways. Using human lung fibroblast MRC-5 cells, we demonstrated that SK-216 inhibited TGF-β-dependent differentiation of fibroblasts to myofibroblasts. We also observed this inhibition by SK-216 in human primary lung fibroblasts. Following these *in vitro* results, we tested oral administration of SK-216 into mice injected intratracheally with bleomycin. We found that SK-216 reduced the degree of bleomycin-induced pulmonary fibrosis in mice. Although the precise mechanisms underlying the link between TGF-β and PAI-1 regarding fibrotic process were not determined, PAI-1 seems to act as a potent downstream effector on the pro-fibrotic property of TGF-β. In addition, inhibition of PAI-1 activity by a PAI-1 inhibitor exerts an antifibrotic effect even *in vivo*. These data suggest that targeting PAI-1 as a downstream effector of TGF-β could be a promising therapeutic strategy for pulmonary fibrosis.

## Introduction

Pulmonary fibrosis is a lung disease that includes a heterogeneous group of lung disorders characterized by irreversible destruction of lung architecture. Idiopathic pulmonary fibrosis (IPF), a particularly severe form of pulmonary fibrosis with unknown etiology, features a progressive fibrotic process [1, 2], with no effective strategy to control progression [3].

Although many factors have been proposed to be involved in the development and progression of pulmonary fibrosis, it is widely accepted that transforming growth factor β (TGF-β) plays a central role. TGF-β acts as a major pro-fibrotic cytokine that induces fibroblast migration, proliferation and differentiation of myofibroblasts, and deposition of extracellular matrix [4, 5]. TGF-β is also known to strongly induce epithelial-mesenchymal transition (EMT) that contributes to the generation and accumulation of fibroblasts and myofibroblasts responsible for excessive extracellular matrix deposition [6, 7, 8]. This process is accompanied by the loss of intercellular cohesion and epithelial makers, such as E-cadherin, and the acquisition of spindle-shape, motile, and mesenchymal markers, such as vimentin or fibronectin, in epithelial cells [8–11]. Although there exists strong evidence for TGF-β in this process, little progress has been made in the context of using TGF-β inhibitors for the treatment of pulmonary fibrosis [12].

Plasminogen activator inhibitor-1 (PAI-1) is a member of the serine protease inhibitor family [13]. The primary role of PAI-1 is the regulation of plasminogen activator system through its inhibitory action. The role of PAI-1 in lung fibrotic processes has also been firmly established [14–17]. It has been shown that overexpression of the PAI-1 gene enhances bleomycin (BLM)-induced pulmonary fibrosis [15], and mice deficient in the PAI-1 gene are protected from BLM-induced pulmonary fibrosis [15, 16]. In addition, intrapulmonary administration of siRNA against PAI-1 limits the development of pulmonary fibrosis in mice after BLM injury [18]. Interestingly, TGF-β is a potent inducer of PAI-1 expression in multiple cell types [19–21]. A recent study demonstrated that oral administration of a PAI-1 inhibitor reduced the degree of pulmonary fibrosis induced by intrapulmonary administration of adenovirus vector encoding TGF-β [22]. Our laboratory has also demonstrated that knockdown of PAI-1 prevented EMT induced by TGF-β in mouse lung epithelial cells [18]. These observations prompted us to speculate that pro-fibrotic properties of TGF-β could be mediated by PAI-1; and consequently, inhibition of PAI-1 could be a novel strategy to counteract TGF-β activity. We investigated the effect of PAI-1 inhibition using a novel small molecule PAI-1 inhibitor, SK-216, to study EMT in alveolar epithelial cells and the differentiation of lung fibroblast cells into myofibroblasts. Both these processes are induced by TGF-β. In addition, we also investigated potential anti-fibrotic activity of SK-216 in a mouse model of BLM-induced pulmonary fibrosis.

## Materials and Methods

### Materials

PAI-1 inhibitor, SK-216 ([5-[[6-[5-(1,1-Dimethylethyl)-2-benzoxazolyl]-2-naphthalenyl]oxy]pentyl] propanedioate) was supplied by Shizuoka Coffein Co., Ltd. (Shizuoka, Japan). The inhibitory activity of SK-216 on PAI-1 was investigated using previously published methods and the IC_50_ was determined to be 44 μmol/L as reported in international patent WO04/010996. Human lung adenocarcinoma epithelial cells (A549), mouse lung epithelial cells (LA-4), and human lung fibroblast cells (MRC-5) were purchased from the Japanese Collection of Research Bioresources Cell Bank (Tokyo, Japan). Human primary lung fibroblasts derived from interstitial lung disease tissue (ILD-derived human primary lung fibroblasts) were established from lung tissues from a 67-year-old female who underwent lung biopsy to diagnose the type of ILD. Histologically, this patient was diagnosed with chronic hypersensitivity pneumonitis. Normal human primary lung fibroblasts were established from a histologically normal section of lung tissues from a 56-year-old male who underwent thoracic surgery for lung cancer. The procedure for obtaining lung tissue cells was approved by the medical ethics committee of Hiroshima University, Japan (Permit Number: 33), and written informed consent was obtained from study participants. The morphologic features and fluorescent images of cells were analyzed using the BZ-9000 fluorescence imaging system (Keyence, Osaka, Japan).

### Cell culture and treatment

A549 cells were seeded at a density of 5×10^3^ cells/well in 8-well Lab-Tek chamber slides for morphologic analysis and immunofluorescence staining, 12×10^3^ cells/well in 24-well plates for reverse transcription polymerase chain reaction (RT-PCR), 2×10^4^ cells/well in 24-well plates for measurement of PAI-1 expression, and 1×10^5^ or 3×10^5^ cells/well in 6-well plates for western blot analysis and soluble collagen analysis. LA-4 cells were seeded at a density of 4×10^4^ cells/well in 24-well plates for RT-PCR. The cells were cultured in Dulbecco's modified Eagle medium (DMEM, Life Technologies, Carlsbad, CA) supplemented with 10% fetal calf serum (FCS) for 48 hours. Thereafter, the cells were pre-incubated with or without SK-216 (50, 100 μM) in serum-free medium for 1 hour followed by stimulation with TGF-β1 (human recombinant TGF-β1, 5 ng/mL, R&D Systems, Minneapolis, MN). Time points for sampling in each experiment are described in the figure legends.

MRC-5 cells and human primary fibroblasts were seeded at a density of 12×10^3^ cells/well in 8-well Lab-Tek chamber slides for immunofluorescence staining, 4×10^4^ cells/well in 24-well plates for real-time quantitative RT-PCR, and 3×10^5^ cells/well in 6-well plates for western blot analysis. After the incubation in DMEM containing 10% FCS for 24 hours, the cells were maintained in serum-free medium for 24 hours. Thereafter, the cells were pre-incubated with or without SK-216 (50, 150 μM) in serum-free medium for 1 hour followed by stimulation with TGF-β1 (5ng/ml). Time points for sampling in each experiment are described in the figure legends.

### Real-time quantitative RT-PCR

Total cellular RNA was extracted from cultured cells or lung tissues of mice using the RNeasy Mini Kit (Qiagen, Valencia, CA) according to the manufacturer's protocol. After extraction, total RNA was converted into cDNA by reverse transcription reaction, and real-time quantitative RT-PCR was performed using the ABI 7500 Fast Real-Time PCR system (Applied Biosystems, Foster City, CA). mRNA expression levels were evaluated and normalized to β-actin as an endogenous reference. Primers used were as follows: E-cadherin (TaqMan Gene Expression Assay ID Hs01023894_m1, Applied Biosystems); vimentin (Hs00185584_m1); N-cadherin (Hs00983056_m1); type I collagen α1 chain (COL1A1, Hs00164004_m1, Mm00801666_g1); fibronectin (Hs01549976_m1, Mm0069266_m1); Snail, (Hs00195591_m1); Slug (Hs00161904_m1); α-smooth muscle actin (α-SMA, Hs00426835_g1, Mm00725412_s1); PAI-1 (HS 01126606_m1, Mm00435858_m1); β-actin (4352935E, 4352341E).

### Immunofluorescence staining

For the detection of E-cadherin or α-SMA, cells were fixed in ice-cold methanol for 10 min and treated with a rabbit polyclonal antibody against E-cadherin (1:150; sc-7870; Santa Cruz Biotechnology, Santa Cruz, CA) or α-SMA (1:100; ab5694; Abcam, Cambridge, UK) for 1 hour. The bound primary antibodies were detected using Zenon Alexa Fluor 488 (1:500; Life Technologies). For the detection of vimentin, cells were fixed in 4% paraformaldehyde for 15 min and permeabilized with phosphate-buffered saline (PBS) containing 0.1% TritonX-100 for 5 min. Thereafter, the cells were exposed to mouse Anti-vimentin eFluor 615 (1:100; eBioscience, San Diego, CA) for 1 hour. Nuclei were stained with 4',6-diamidino-2-phenylindole (DAPI, Life Technologies). Digital images were captured on the at least six fields per sample with the same exposure time for each marker. The degrees of expression for E-cadherin, α-SMA, and vimentin were quantified by measuring fluorescence intensities of Alexa Fluor 488 or eFluor 615 per each field using software (BZ-analysis application, Keyence).

### Measurement of PAI-1 concentration and activity

PAI-1 levels in the supernatant of cell cultures or bronchoalveolar lavage fluid (BALF) were measured using human total, human active, or mouse active PAI-1 ELISA kit (Molecular Innovations, Novi, MI).

### Assessment of endogenous TGF-β production in A549 cells

A549 cells were seeded at a density of 3×10^4^ cells/well in 24-well plates and cultured in DMEM with 10% FCS for 48 hours. Thereafter, the cells were pre-incubated with or without SK-216 (100 μM) in serum-free medium for 1 hour, and then stimulated with TGF-β1 (5ng/mL). After treatment for 24 hours, culture mediums were replaced with serum-free medium one more time. The concentrations of TGF-β1 in the supernatants of the cultures were measured at 12, 24, and 48 hours later using a TGF-β1 ELISA kit (R&D systems, Minneapolis, MN).

### Measurement of soluble collagen

Total soluble collagen in the cell culture supernatant was measured using the Sircol assay kit (Biocolor, Newtownabbey, United Kingdom) following the manufacturer’s instructions. To minimize the influence of cell number, the level of soluble collagen was normalized to total protein in the cell lysate measured using a BCA assay kit (Pierce Biotechnology, Rockford, IL).

### Proliferation assay

The effects of SK-216 in the presence and absence of TGF-β1 on the cell proliferation were analyzed using the cell counting kit-8 (DOJINDO, Kumamoto, Japan) following the manufacturer’s instructions.

### Western blotting

Cells were lysed with Nonidet P-40 (NP-40) lysis buffer (50 mM Tris–HCl [pH 8.0], 150 mM NaCl, 1% NP-40) containing protease inhibitor cocktail. Proteins were separated on sodium dodecyl sulfate‑polyacrylamide gel electrophoresis gels and were electroblotted onto polyvinylidene fluoride membrane (GE Healthcare Bio-sciences, Piscataway, NJ). The polyvinylidene fluoride membranes were incubated with anti-phospho-Smad2 antibody (Ser465/467), anti-phospho-extracellular-signal-regulated kinases (ERK) 1/2 monoclonal antibody (Thr202/Tyr204), anti-total-Smad2 mouse monoclonal antibody, anti-total-ERK1/2 rabbit monoclonal antibody, anti-E-cadherin rabbit monoclonal antibody, anti-vimentin rabbit monoclonal antibody, anti-N-cadherin rabbit monoclonal antibody (Cell Signaling, Danvers, MA), anti-α-SMA rabbit polyclonal antibody, anti-collagen I rabbit polyclonal antibody (Abcam), or anti-fibronectin mouse monoclonal antibody (BD Biosciences). The HRP‑conjugated goat anti-rabbit or anti-mouse IgG (GE Healthcare Bio-sciences) served as the secondary antibodies. The immunolabeled proteins were visualized by enhanced chemiluminescence. The band intensity was analyzed densitometrically by using ImageJ software (NIH).

### Bleomycin exposure and oral administration of SK-216

Female C57BL/6 mice were purchased from Charles River Laboratories (Kanagawa, Japan). All animal experiments were approved by the animal ethics committee of Hiroshima University, Japan (Permit Number: A10-100). All efforts were made to minimize animal suffering, including anesthesia by using an intraperitoneal injection with pentobarbital. Mice were monitored daily for signs of distress and changes in body weight. Mice were randomly allocated into three weight-matched groups (n = 6 each group): PBS+DW group, PBS intratracheally instilled and orally administered with distilled water; BLM+DW group, BLM intratracheally instilled and orally administered with distilled water; BLM+SK-216 group, BLM intratracheally instilled and orally administered with distilled water containing SK-216. On day 0, after anesthesia with pentobarbital, mice were intratracheally instilled with PBS or PBS containing BLM (1.5) mg/kg body weight, Nippon Kayaku, Tokyo, Japan) as previously described [18]. For the BLM+SK-216 group, the mice were administered with distilled water on days from 0 to 8 and were given distilled water containing SK-216 (1000ppm) on days from 9 to 21. Mice were sacrificed on day 11 or 21 under deep anesthesia with pentobarbital for euthanasia.

### Assessment of pulmonary fibrosis in mice

On day 11 after bleomycin injection, BALF was obtained as previously described [18] and lungs were harvested for real-time quantitative RT-PCR. On day 21 after bleomycin injection, lungs were harvested for hydroxyproline assay and histological examinations. The degree of pulmonary fibrosis was determined by measuring hydroxyproline content in whole lung tissue as previously described [16]. After perfusion with PBS, the right lungs were fixed with 4% paraformaldehyde and embedded in paraffin for histological examinations. The sections were stained with hematoxylin and eosin (H&E) or Masson’s trichrome. Furthermore, for detection of α-SMA expression in tissue cells, immunohistochemical staining with mouse anti-α-SMA antibody (1/500 dilution, DAKO, Denmark) was performed using a Histofine Mousestain Kit (Nichirei, Tokyo, Japan). The immunoreaction was visualized by incubation with diaminobenzidine (DAB) chromophoric solution. The sections were then counterstained with Mayer’s hematoxylin.

### Statistical analyses

Data are expressed as the mean ± SEM. The statistical significances were analyzed by the Mann–Whitney U test or Student's t-test using SPSS software version 19 (SPSS Japan, Tokyo, Japan). A p value of <0.05 was considered to be statistically significant.

## Results

### Effect of SK-216 on TGF-β-induced EMT

**Fig 1:**
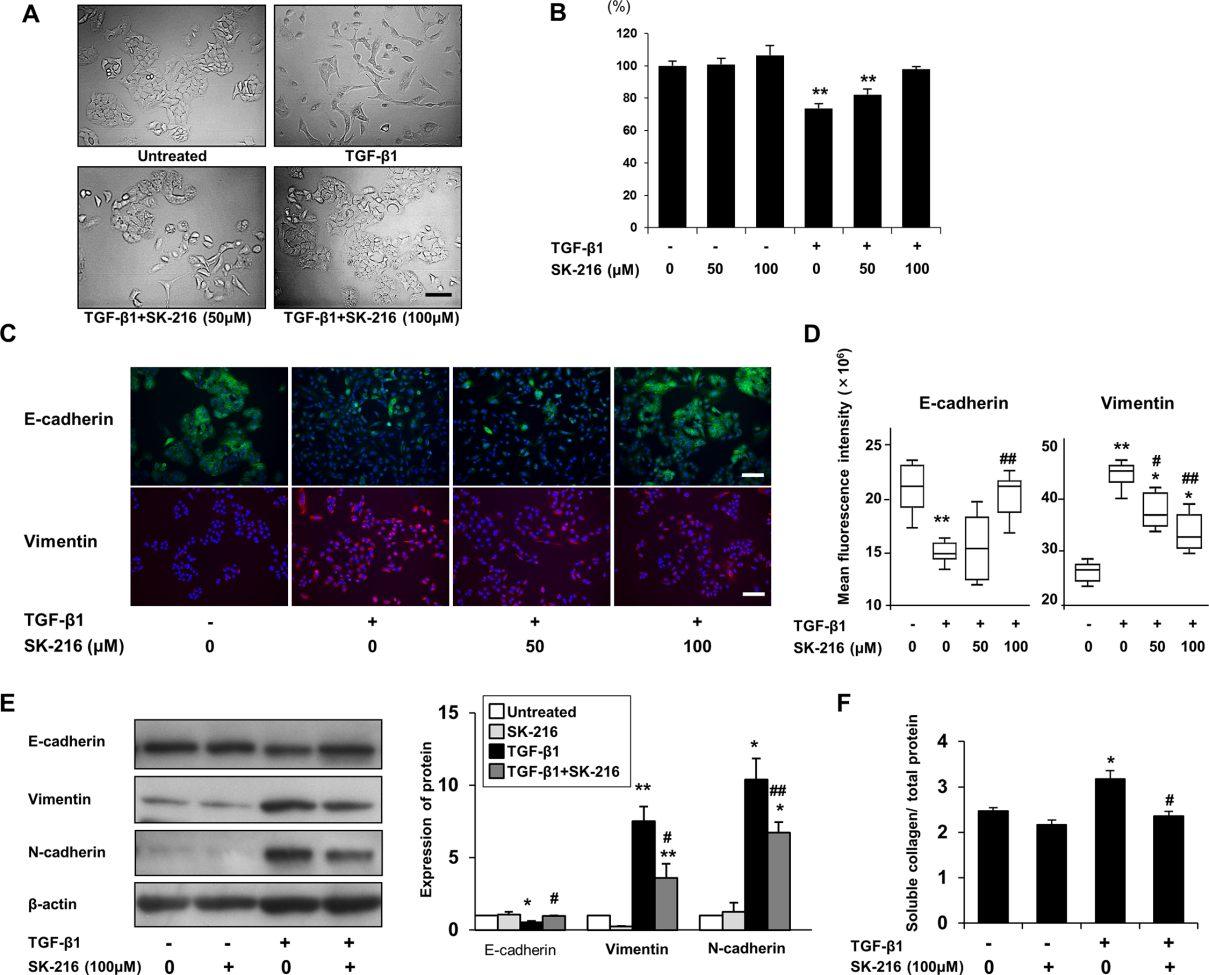
Effect of SK-216 on morphological transformation and the expression of epithelial or mesenchymal markers in A549 cells undergoing TGF-β-induced EMT. Cells were pre-incubated in the presence or absence of SK-216 (50, 100 μM) for 1 hour and then co-incubated with TGF-β1 (5 ng/ml). All assays were performed after 48 hours of incubation. (A) Phase contrast images of A549 cells. A549 cells untreated with TGF-β1 maintained epithelial cobblestone-shape with cell-cell contacts. The treatment with TGF-β1 transformed the cells into mesenchymal spindle-shape cells with loss of cell-cell adhesion. The co-incubation with SK-216 reversed these TGF-β-induced morphologic alterations. Magnification: ×200. Scale bar = 100 μm. (B) Proliferation of A549 cells after treatment with SK-216 and TGF-β1. (C) A549 cells stained for E-cadherin (green), vimentin (red) and nuclei (blue). Magnification: ×200. Scale bar = 100 μm. (D) Fluorescence intensities for E-cadherin and vimentin. Results are expressed as Box plot of at least 6 fields. (E) Western blot analysis of E-cadherin, vimentin, and N-cadherin. Data normalized to β-actin levels are shown as fold change from the control, and reflect the mean ± SEM of 3 independent experiments. (F) Measurement of total soluble collagen in the culture medium. Data are normalized to the amount of total protein in the cell lysate. Results are expressed as the mean ± SEM of 4 independent experiments. *p <0.05, **p <0.01 vs untreated cells, ^#^p <0.05, ^##^p <0.01 vs cells treated with TGF-β1.

**Fig 2:**
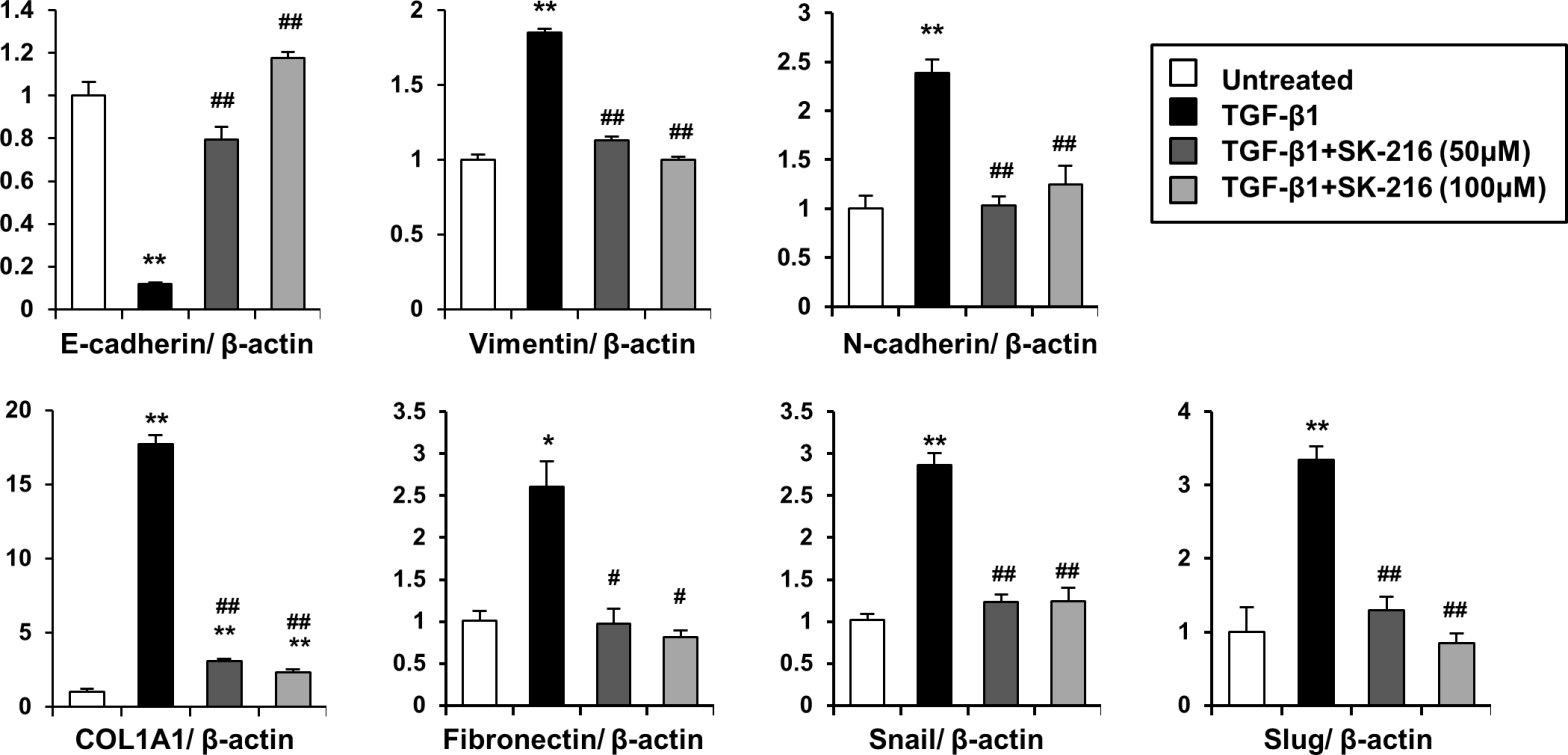
Effect of SK-216 on mRNA expression of epithelial or mesenchymal markers in A549 cells undergoing TGF-β-induced EMT. The cells were pre-incubated in the presence or absence of SK-216 (50, 100 μM) for 1 hour and then co-incubated with TGF-β1 (5 ng/ml). After incubation for 24 hours, the cells were harvested and total RNA was extracted. The mRNA expression shows E-cadherin as an epithelial marker; vimentin, N-cadherin, COL1A1, and fibronectin as mesenchymal markers; and EMT-related transcription factors, Snail and Slug using real-time quantitative RT-PCR. Results are expressed as fold change from the control and reflect the mean ± SEM of 4–5 experiments. *p <0.05, **p <0.01 vs untreated cells, ^#^p <0.05, ^##^p <0.01 vs cells treated with TGF-β1.

To determine whether PAI-1 is involved in TGF-β-induced EMT, we treated A549 cells with TGF-β1 in the presence or absence of SK-216 and assessed the effects of SK-216 on changes in morphology and expression patterns of EMT-related markers. As shown in Fig 1A, the treatment of A549 cells with TGF-β1 transformed cobblestone-shaped epithelial cells into spindle-shaped mesenchymal cells with loss of cell-cell contacts. However, the co-presence of SK-216 with TGF-β1 in the culture was shown to maintain epithelial cell like morphology of A549 cells. We performed a cell proliferation assay to evaluate the influence of SK-216 on the viability of A549 cells. SK-216 did not affect the proliferation of A549 cells in the absence of TGF-β1 but reversed the TGF-β-induced suppression in proliferation of A549 cells (Fig 1B). Next, we investigated the relationship between these morphological changes and expression patterns of epithelial and mesenchymal markers in A549 cells. As shown in Fig 1C, TGF-β1 treatment decreased the expression level of E-cadherin and increased vimentin levels; however the co-presence of SK-216 with TGF-β1 counteracted the decrease in E-cadherin expression level and the increase in vimentin expression level. Fluorescence quantification showed that SK-216 restored the E-cadherin expression level eliminated by TGF-β1 and reduced the vimentin expression level elevated by TGF-β1 (Fig 1D). We also analyzed the protein levels of EMT-related markers in A549 cells using western blotting. As shown in Fig 1E, SK-216 reduced the decrease of E-cadherin and the increase in vimentin and N-cadherin in TGF-β1-treated A549 cells. In addition, SK-216 was found to inhibit the TGF-β-induced increase of soluble collagen in the culture supernatants of A549 cells (Fig 1F). We next tested mRNA expression levels of EMT-related markers (E-cadherin, vimentin, N-cadherin), transcription factors (Snail and Slug), and extracellular matrices (type I collagen and fibronectin) in A549 cells under the same conditions. As shown in Fig 2, TGF-β1 treatment downregulated mRNA levels of E-cadherin and upregulated mRNA levels of vimentin, N-cadherin, type I collagen α1 chain (COL1A1), fibronectin, Snail, and Slug. However, the co-presence of SK-216 with TGF-β1 significantly reversed these changes.

### Effect of SK-216 on TGF-β-induced PAI-1 expression

**Fig 3:**
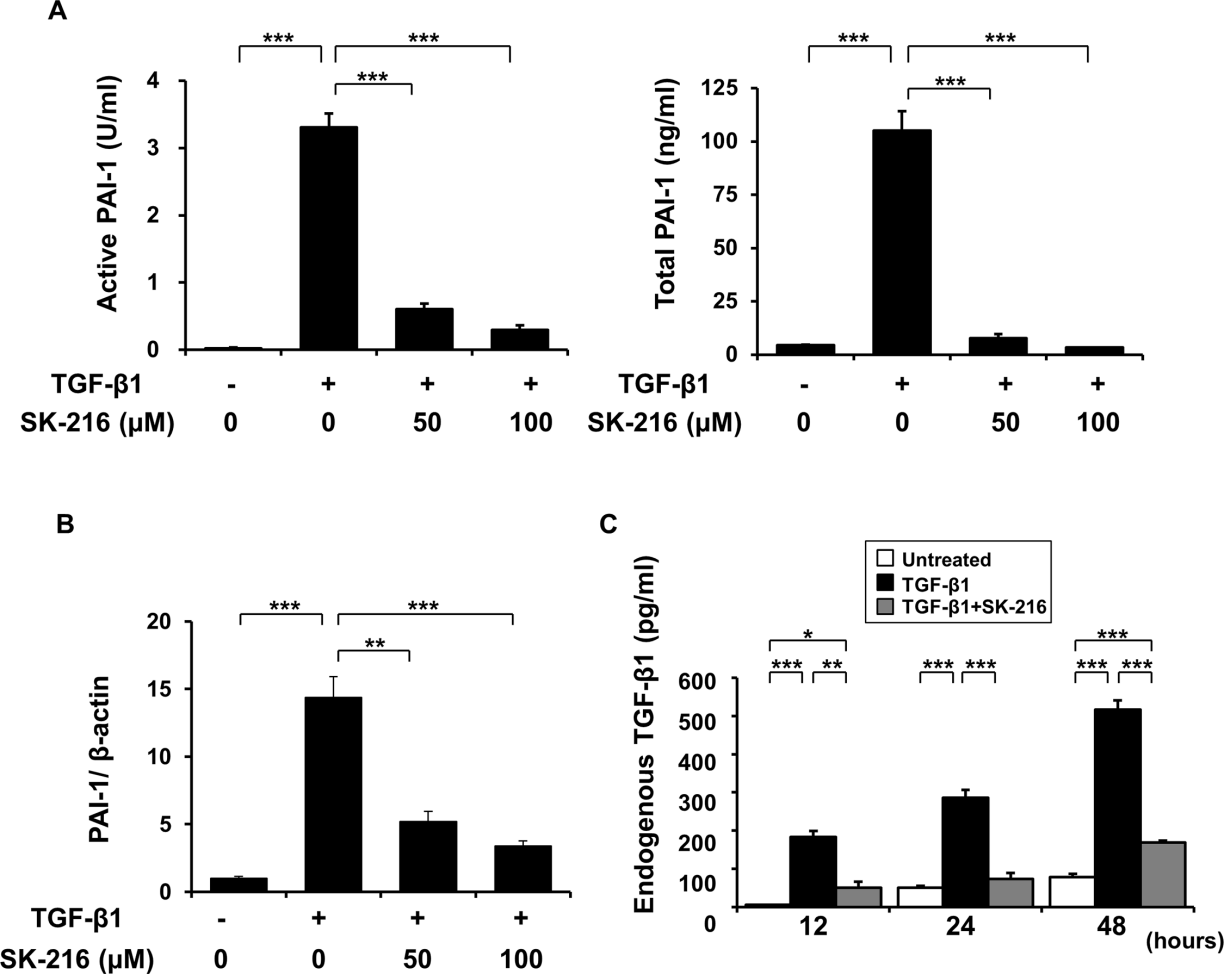
PAI-1 expression (A-B) and production of endogenous TGF-β1 (C) in A549 cells when TGF-β-induced EMT was blocked by SK-216. (A-B) The cells were pre-incubated in the presence or absence of SK-216 (50, 100 μM) for 1 hour and then co-incubated with TGF-β1 (5 ng/ml). After incubation for 24 hours, the cells and the supernatants of cell cultures were collected. (A) Active and total PAI-1 levels in the supernatants were measured using active and total PAI-1 ELISA kit. Results are expressed as the mean ± SEM of 4 samples. (B) PAI-1 mRNA expression in the cells was measured using real-time quantitative RT-PCR. Results are expressed as the mean ± SEM of 5 experiments. (C) The cells were pre-incubated in the presence or absence of SK-216 (100 μM) for 1 hour and then co-incubated with TGF-β1 (5 ng/ml). After incubation for 24 hours, the culture medium was replaced with serum-free medium. The supernatants of cell cultures were collected at 12, 24, and 48 hours after the medium change. Endogenous TGF-β1 was analyzed by measuring the concentration of TGF-β1 in the supernatants using TGF-β1 ELISA kit. Results are expressed as the mean ± SEM of 4 experiments. *p < 0.05, **p < 0.01, ***p < 0.001.

TGF-β is a potent inducer of PAI-1 expression in various types of cells [19–21]. To assess whether TGF-β induces PAI-1 expression in A549 cells even when EMT is being blocked by SK-216, we evaluated PAI-1 concentration in the culture supernatants and mRNA expression level of PAI-1 in these cells. As shown in Fig 3A and 3B, TGF-β1 stimulation significantly increased PAI-1 levels in the supernatants and PAI-1 mRNA expression in A549 cells. However, the co-presence of SK-216 with TGF-β1 in the culture inhibited the elevation of PAI-1 levels in the culture supernatants and PAI-1 mRNA expression levels in the cells.

### Production of endogenous TGF-β1 in A549 cells upon TGF-β-induced EMT blocked by SK-216

We next investigated whether production of endogenous TGF-β was enhanced following TGF-β-induced EMT in A549 cells and if this production was affected by SK-216. As shown in Fig 3C, the production of endogenous TGF-β1 was markedly elevated at 12, 24, and 48 hours after exogenous TGF-β1 stimulation. However, the co-treatment of A549 cells with SK-216 and TGF-β1 significantly suppressed these elevations.

### Effect of SK-216 on TGF-β-induced Smad and ERK phosphorylation in A549 cells

**Fig 4:**
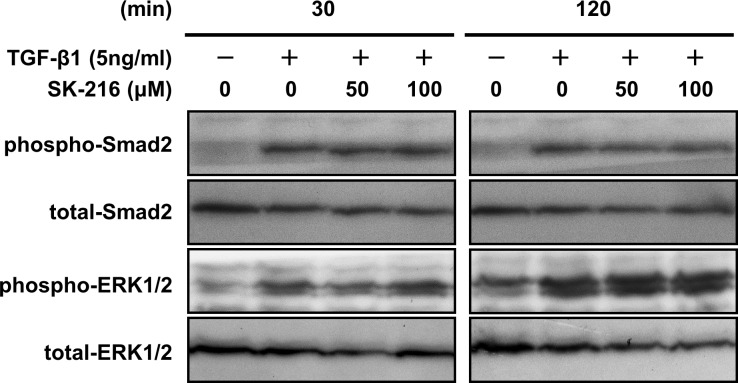
Effect of SK-216 on TGF-β-induced Smad2 and ERK1/2 phosphorylation in A549 cells. A549 cells were pre-incubated in the presence or absence of SK-216 (50, 100 μM) for 1 hour and then co-incubated with TGF-β1 (5 ng/ml) for 30 and 120 min. The cell lysates were subjected to western blotting with antibody against Smad2, phosphorylated Smad2, ERK1/2, and phosphorylated ERK1/2.

Smad and ERK signaling are two important pathways for TGF-β-induced EMT [23–25]. To assess the mechanism by which SK-216 blocks TGF-β-induced EMT, we evaluated the effects of SK-216 on Smad2 and ERK1/2 phosphorylation in A549 cells undergoing EMT. As shown in Fig 4, Smad2 and ERK1/2 were phosphorylated after the stimulation with TGF-β1 for 30 and 120 min. However, the co-presence of SK-216 with TGF-β1 did not inhibit these phosphorylations. This indicates that SK-216 inhibits TGF-β-induced EMT independent of these pathways.

### Effect of SK-216 on TGF-β-induced EMT in LA-4 cells

**Fig 5:**
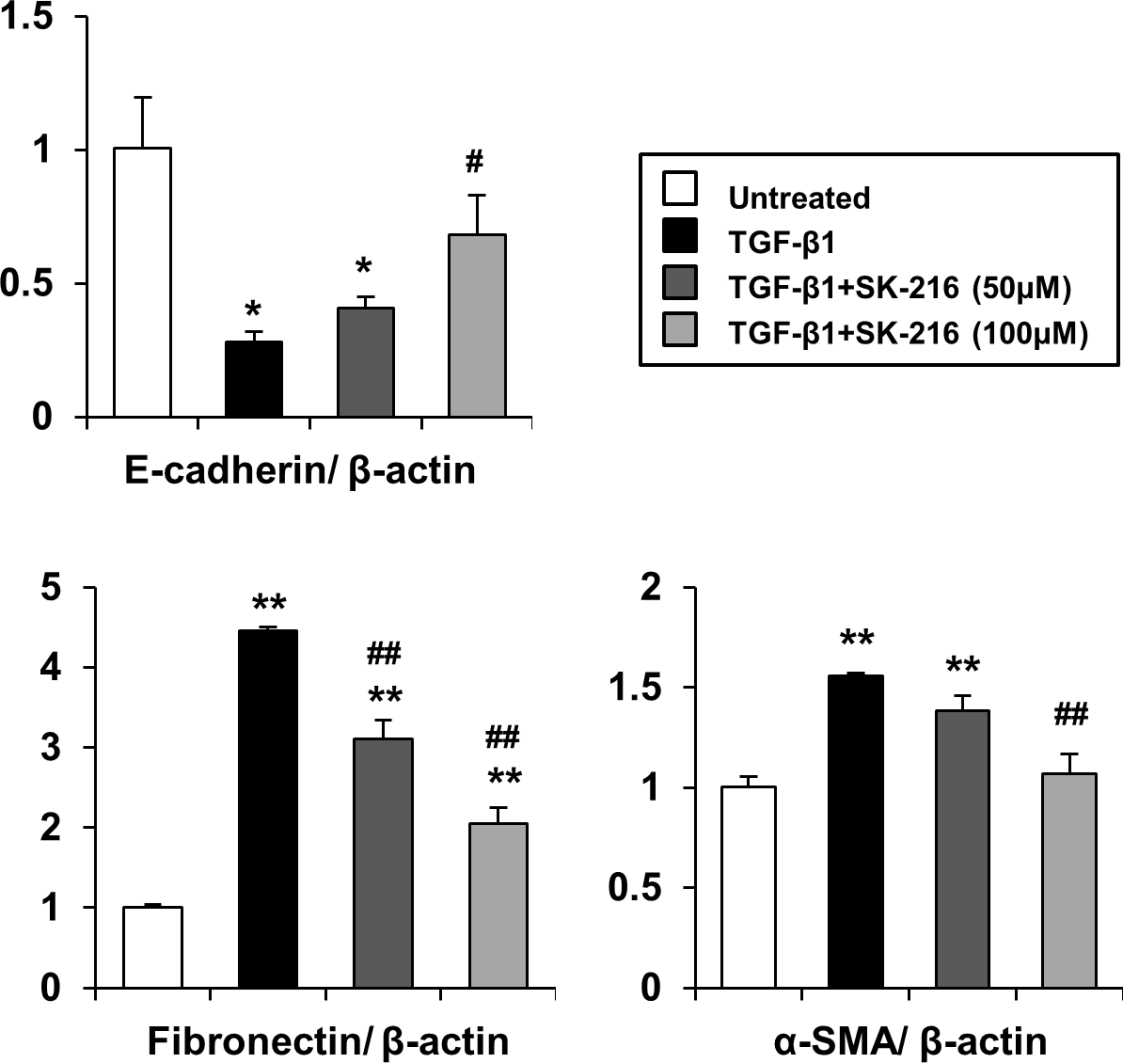
Effect of SK-216 on mRNA expression of epithelial or mesenchymal markers in LA-4 cells undergoing TGF-β-induced EMT. The cells were pre-incubated in the presence or absence of SK-216 (50, 100 μM) for 1 hour and then co-incubated with TGF-β1 (5 ng/ml). After incubation for 24 hours, the cells were harvested and total RNA was extracted. The mRNA expression shows E-cadherin as an epithelial marker, and fibronectin and α-SMA as mesenchymal markers using real-time quantitative RT-PCR. Results are expressed as fold change from the control and reflect the mean ± SEM of 4 experiments. *p <0.05, **p <0.01 vs untreated cells, ^#^p <0.05, ^##^p <0.01 vs cells treated with TGF-β1.

To confirm the effect of SK-216 on TGF-β-induced EMT in other alveolar epithelial cells, we conducted an experiment using mouse lung epithelial LA-4 cells. As shown in Fig 5, the stimulation with TGF-β1 downregulated E-cadherin mRNA expression and upregulated fibronectin and α-SMA mRNA expression. However, the co-presence of SK-216 with TGF-β1 in the culture reversed these changes. These results indicate that SK-216 also inhibits TGF-β-induced EMT in LA-4 cells. Vimentin was not used as an EMT-related marker for LA-4 cells because TGF-β1 was found to decrease its mRNA levels (data not shown).

### Effect of SK-216 on TGF-β-induced differentiation of fibroblasts to myofibroblasts

**Fig 6:**
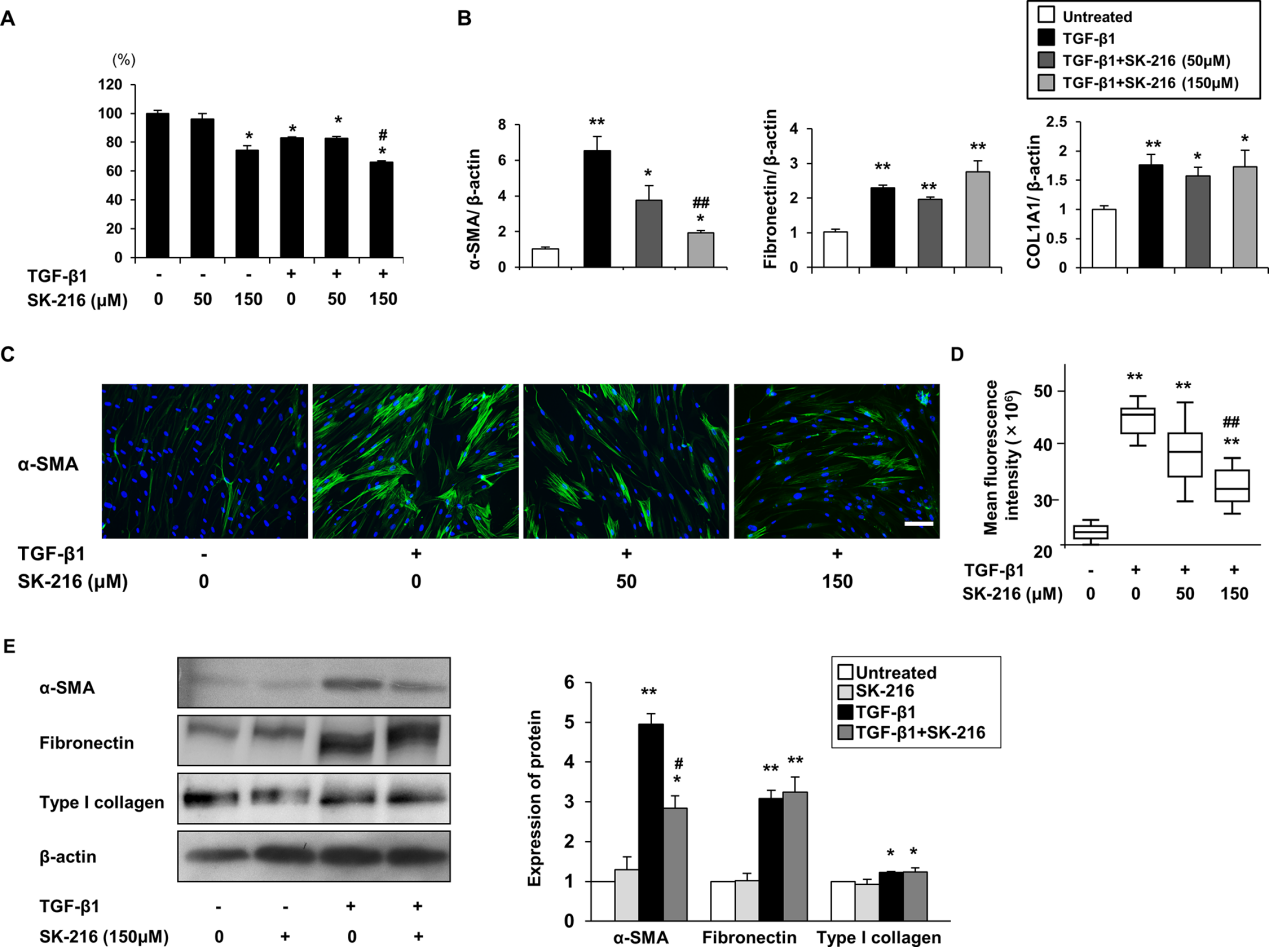
Effect of SK-216 on fibroblast to myofibroblast differentiation induced by TGF-β1 in MRC-5 cells. After pre-incubation in serum free medium for 24 hours, MRC-5 cells were pretreated in the presence or absence of SK-216 (50, 150 μM) for 1 hour followed by stimulation with TGF-β1 (5 ng/ml). Real-time quantitative RT-PCR analysis was performed after incubation for 24 hours. Proliferation assay, immunofluorescence staining, and western blotting analysis were performed after incubation for 48 hours. (A) Proliferation of MRC-5 cells after treatment with SK-216 and TGF-β1. (B) mRNA expression of α-SMA, fibronectin, and COL1A1 in MRC-5 cells evaluated using real-time quantitative RT-PCR. Results are expressed as fold change from the control and reflect the mean ± SEM of 4–5 experiments. (C) MRC-5 cells stained for α-SMA labeled with Zenon Alexa Fluor 488 (green) and nucleus labeled with DAPI (blue). Magnification: ×400. Scale bar = 50 μm. (D) Fluorescence intensities for α-SMA in MRC-5 cells. Results are expressed as Box plot of at least 6 fields. (E) Western blot analysis of α-SMA, fibronectin, and type I collagen. Data normalized to β-actin levels are shown as fold change from the control and reflect the mean ± SEM of 3 independent experiments. *p <0.05, **p <0.01 vs untreated cells, ^#^p <0.05, ^##^p <0.01 vs cells treated with TGF-β1.

**Fig 7:**
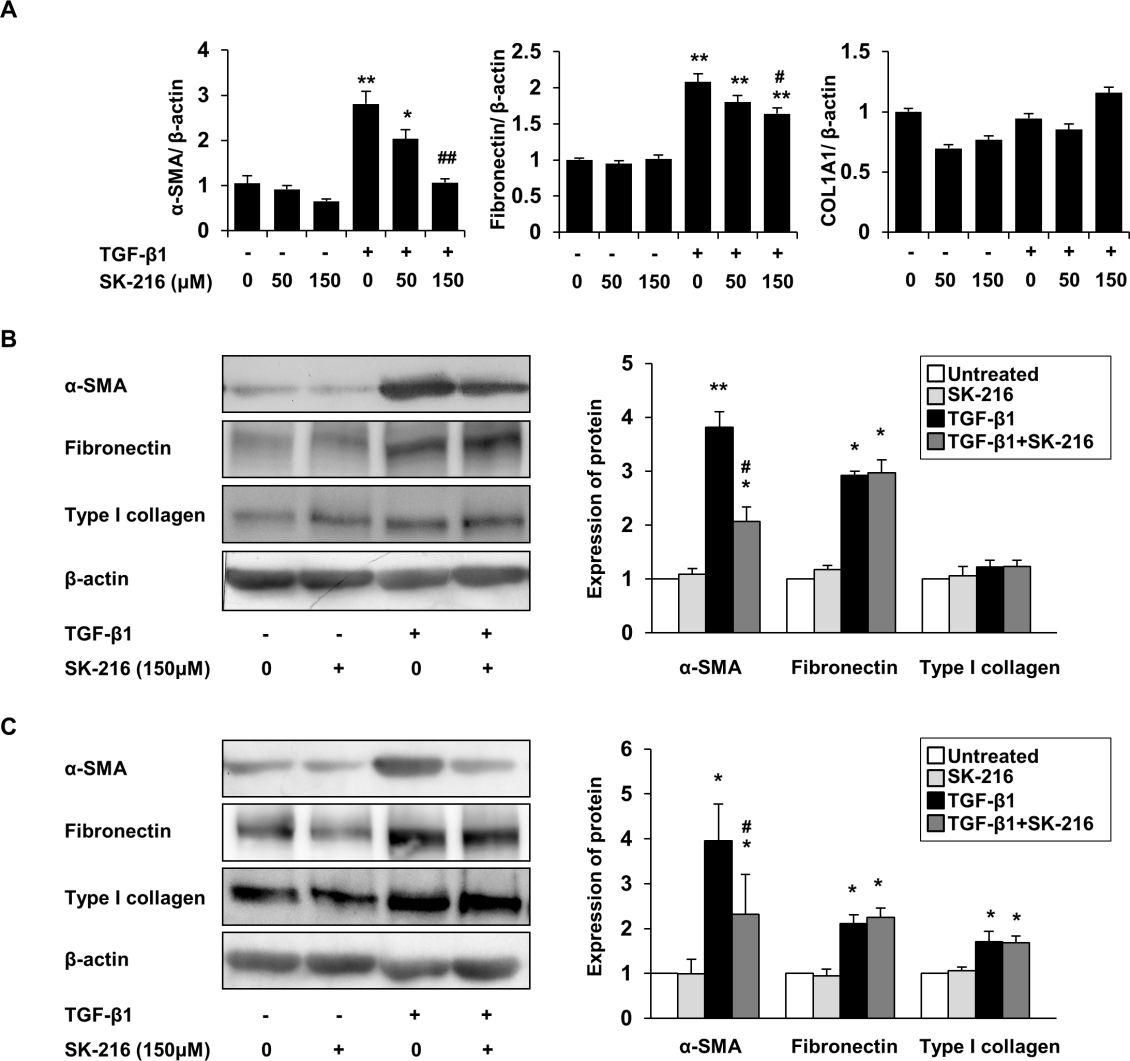
Effect of SK-216 on fibroblast to myofibroblast differentiation induced by TGF-β1 in human primary lung fibroblasts. After pre-incubation in serum free medium for 24 hours, ILD-derived and normal human primary lung fibroblasts were pretreated in the presence or absence of SK-216 (50, 150 μM) for 1 hour followed by stimulation with TGF-β1 (5 ng/ml). Real-time quantitative RT-PCR analysis and western blotting analysis were performed after incubation for 24 and 48 hours, respectively. (A) mRNA expression of α-SMA, fibronectin, and COL1A1 in ILD-derived human primary lung fibroblasts evaluated using real-time quantitative RT-PCR. Results are expressed as fold change from the control and reflect the mean ± SEM of 4 experiments. (B-C) Western blot analysis of α-SMA, fibronectin, and type I collagen in (B) ILD-derived and (C) normal human primary lung fibroblasts. Data normalized to β-actin levels are shown as fold change from the control and reflect the mean ± SEM of 3 independent experiments. *p <0.05, **p <0.01 vs untreated cells, ^#^p <0.05, ^##^p <0.01 vs cells treated with TGF-β1.

Differentiation of fibroblasts to myofibroblasts is believed to play an important role in the progression of pulmonary fibrosis, and studies report a role for PAI-1 in this process [22, 26]. To determine whether SK-216 exerts an inhibitory effect on TGF-β-induced differentiation of fibroblasts to myofibroblasts, we evaluated the expression of α-SMA in MRC-5 cells treated with TGF-β1 in the presence or absence of SK-216. The proliferation assay showed that in the presence or absence of TGF-β1, 50 μM of SK-216 did not affect cell proliferation, but 150 μM of SK-216 was able to suppress the proliferation of MRC-5 cells (Fig 6A). RT-PCR analysis showed that TGF-β1 treatment increased the mRNA level of α-SMA in MRC-5 cells; whereas co-treatment of MRC-5 cells with TGF-β1 and SK-216 significantly reversed this increase (Fig 6B). Furthermore, immunofluorescence staining for α-SMA demonstrated that the treatment of MRC-5 cells with TGF-β1 enhanced the intensity of α-SMA staining and the co-treatment with SK-216 blocked this enhancement (Fig 6C). Quantification of fluorescence intensity revealed that SK-216 significantly reduced TGF-β-induced expression of α-SMA (Fig 6D). Consistent with the results of RT-PCR and immunofluorescence staining, western blotting analysis demonstrated that co-treatment with SK-216 significantly reduced TGF-β-induced expression of α-SMA (Fig 6E). We also evaluated the effect of SK-216 on fibronectin and type I collagen expression in MRC-5 cells treated with TGF-β1. In contrast to its effect on α-SMA expression, SK-216 did not affect TGF-β-induced increases in fibronectin or type I collagen expression at either the mRNA or protein level (Fig 6B and 6E). To examine whether SK-216 inhibits TGF-β-induced myofibroblastic differentiation in other lung fibroblasts, we next conducted experiments using ILD-derived and normal human primary lung fibroblasts. As observed in MRC-5 cells, the co-treatment with SK-216 also blocked TGF-β-induced upregulation of α-SMA expression at both the mRNA and protein level in these primary lung fibroblasts (Fig 7A–7C). Meanwhile, SK-216 marginally reduced TGF-β-induced expression of fibronectin mRNA in ILD-derived human primary lung fibroblasts (Fig 7A), but fibronectin protein levels were not affected in either primary lung fibroblast type (Fig 7B and 7C). SK-216 also did not affect either mRNA or protein expression of type I collagen in these primary lung fibroblasts (Fig 7A–7C). Taken together, we can speculate that SK-216 significantly affects α-SMA expression but its effect on extracellular matrix (ECM) production is negligible during fibroblast to myofibroblast differentiation.

### Effect of SK-216 on BLM-induced pulmonary fibrosis

**Fig 8:**
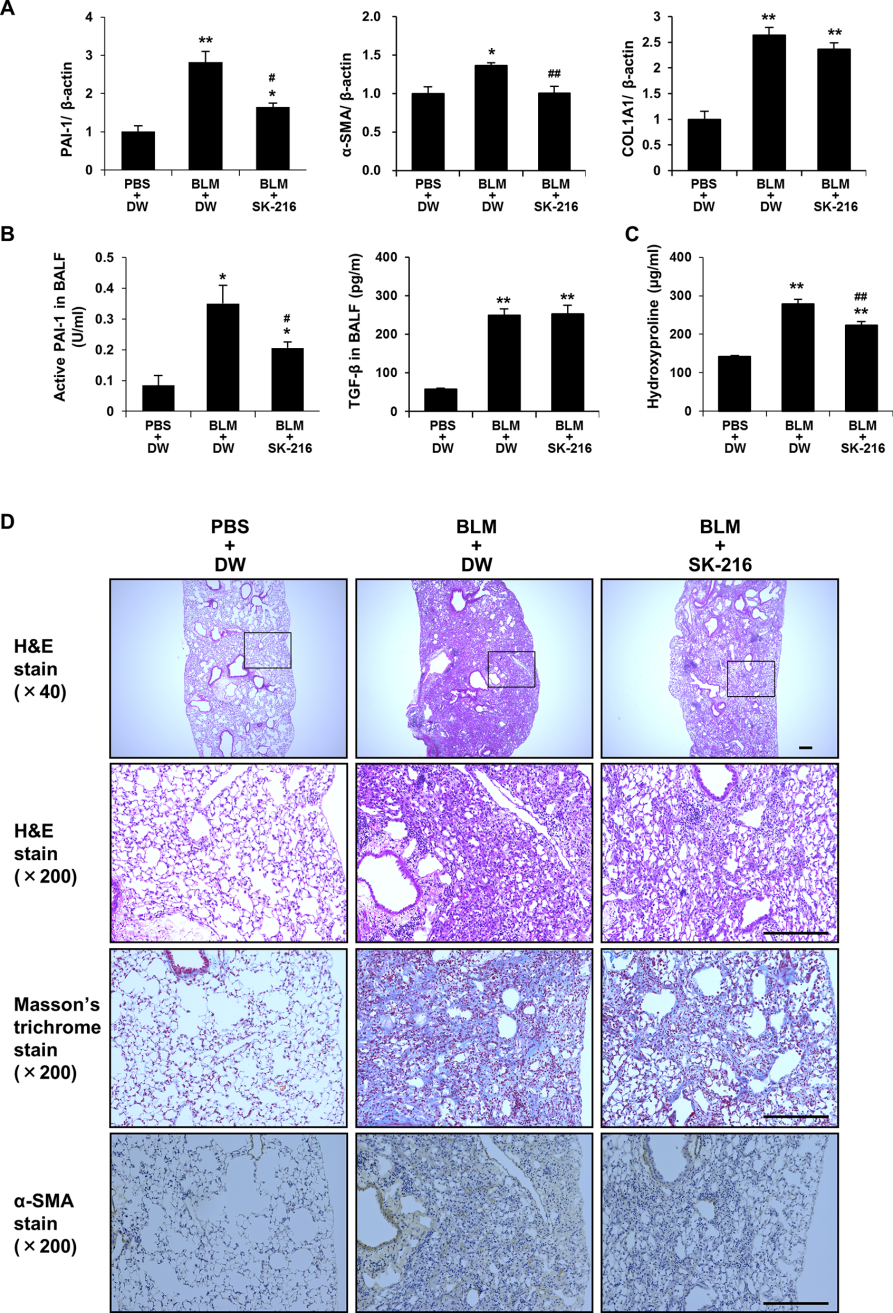
Effect of SK-216 on BLM-induced pulmonary fibrosis in mice. Mice were allocated into three groups: PBS+DW group, BLM+DW group, BLM+SK-216 group. Mice were intratracheally instilled with PBS or PBS containing BLM (1.5 mg/kg body weight) on day 0. PBS+DW group; PBS intratracheally instilled and orally administered with distilled water, BLM+DW group; BLM intratracheally instilled and orally administered with distilled water, BLM+SK-216 group; BLM intratracheally instilled and orally administered with distilled water on days from 0 to 8 and distilled water containing SK-216 (1000ppm) on days from 9 to 21. (A) mRNA expression levels of PAI-1, α-SMA, and COL1A1 in lung. Left lung harvested on day 11 was analyzed using real-time quantitative RT-PCR. Results are expressed as fold change from the control. (B) Levels of active PAI-1 and TGF-β1 in BALF. BALF was collected on day 11. (C) The degrees of pulmonary fibrosis were analyzed by measuring hydroxyproline contents in the whole lungs on day 21. (D) Histological and immunohistochemical analysis of bleomycin-injured lung. Right lungs were excised and sectioned on day 21. Scale bar = 200 μm. Results are presented as the mean ± SEM of 6 mice per group. *p <0.05, **p <0.01 vs PBS+DW group, ^#^p <0.05, ^##^p <0.01 vs BLM+DW group.

Following the results that SK-216 suppressed TGF-β-induced EMT and differentiation of fibroblasts to myofibroblasts *in vitro*, we next investigated whether SK-216 exerts an antifibrotic effect on BLM-induced pulmonary fibrosis *in vivo*. To accurately assess the antifibrotic activity of SK-216 *in vivo*, SK-216 was started at day 9 after BLM injection when the fibrotic phase of BLM-induced pulmonary fibrosis is thought to begin. No mice died during the study period. The expression levels of PAI-1 mRNA in lung tissue and active PAI-1 level in BALF 11 days after BLM injection were significantly higher in BLM+DW mice compared with PBS+DW mice. SK-216 administration reversed these effects (Fig 8A and 8B). Similarly, the expression level of α-SMA mRNAs in lung tissues was also higher in BLM+DW mice compared with PBS+DW mice, whereas the administration of SK-216 was shown to counteract this elevation (Fig 8A). Although SK-216 administration tended to lower type I collagen α1 chain (COL1A1) mRNA levels in lung tissues, the differences between BLM+DW mice and BLM+SK-216 mice did not reach statistical significance (Fig 8A). However, as shown in Fig 8C, the increased hydroxyproline content in the lungs in response to BLM injection was significantly reduced by the administration of SK-216 during the fibrotic phase of BLM-induced pulmonary fibrosis. In contrast to these inhibitory effects of SK-216, TGF-β1 levels in BALF from BLM+SK-216 mice on day 11 did not differ from those of BLM+DW mice (Fig 8B). Histological and immunohistochemical analyses demonstrated that the administration of SK-216 appeared to reduce the extent of fibrotic changes and the degree of collagen deposition and α-SMA expression in BLM-injured mice (Fig 8D). These results show that SK-216 also exerts anti-fibrotic effects *in vivo*.

## Discussion

In this study, we show that SK-216, a PAI-1 specific inhibitor, blocks TGF-β-induced EMT in lung epithelial A549 cells. This inhibition of EMT by SK-216 reduces the TGF-β-stimulated production of PAI-1 and endogenous TGF-β in A549 cells. In addition, SK-216 is effective at inhibiting TGF-β-induced differentiation of MRC-5 lung fibroblasts into myofibroblasts. These *in vitro* results suggest that the biological action of TGF-β in fibrotic processes can be suppressed by inhibition of PAI-1. Furthermore, oral administration of SK-216 to BLM injured mice reduces the degree of pulmonary fibrosis, suggesting that the inhibition of PAI-1 is effective at limiting pulmonary fibrosis *in vivo*.

The most interesting finding of the present study is that the biological effects of TGF-β on EMT and fibroblast to myofibroblast differentiation were blocked by inhibiting PAI-1 using a PAI-1 specific inhibitor. We previously showed that siRNA-mediated knockdown of PAI-1 in LA-4 cells inhibits the induction of EMT by TGF-β [18], suggesting a role for PAI-1 during TGF-β-induced EMT. The results of the present study strengthen the fact that PAI-1 is associated with TGF-β-induced EMT, and further confirm that PAI-1 is a potent downstream effector of TGF-β on EMT and fibroblast to myofibroblast differentiation.

TGF-β-mediated signaling pathways that induce EMT have been well studied. Although the Smad signaling pathway is known to play a key role in TGF-β-induced EMT, non-Smad signaling pathways, including mitogen-activated protein kinases (MAPKs) such as ERKs, the c-junNH2-terminal kinases (JNKs), and the p38 MAPK, the phosphoinositide-3-kinase (PI3K), and AKT have also been reported to mediate TGF-β-induced EMT [23–25]. Zhang et al. showed that, in lung fibroblasts from BLM injured mice, transfection of siRNA against PAI-1 inhibited the phosphorylation of ERK and AKT, whereas overexpression of PAI-1 increased these phosphorylations [26]. It has also been reported that recombinant PAI-1 enhances ERK phosphorylation in mesangial cells [27]. These findings suggest that PAI-1 might activate the ERK signaling pathway. Based on these observations, we examined whether SK-216 would inhibits Smad and/or ERK signaling pathways. However, western blot analysis of extracts from A549 cells treated with TGF-β in the presence or absence of SK-216 revealed that SK-216 did not affect the phosphorylation of Smad2 and ERK1/2. We also found that SK-216 inhibited mRNA expression of Snail and Slug, transcriptional factors strongly associated with EMT. Although we failed to elucidate the molecular mechanisms by which SK-216 blocked TGF-β-induced EMT, the inhibition of Snail and Slug expressions by SK-216 suggests that SK-216 blocked TGF-β-mediated signaling pathways related to EMT other than Smad and ERK. As another possible mechanism, the binding of PAI-1 to vitronectin is known to modulate cell-matrix adhesion, cell migration, and cell phenotype via its interaction with integrins or urokinase-type plasminogen activator receptor (u-PAR) [28–30] and a recent study indicates that the vitronectin-binding function of PAI is more important for the progression of pulmonary fibrosis than its anti-protease activity [31]. In this light, we suspected that SK-216 might block the binding between PAI-1 and vitronectin and thus inhibit EMT. Unfortunately, however, suitable assay systems to test this possibility were not available to us. Further investigation is necessary to better understand the role of PAI-1 in TGF-β-induced EMT.

The differentiation of lung fibroblasts to myofibroblasts, which is characterized by high expression of α-SMA, promotes the aberrant deposition of extracellular matrix and contributes to the development of pulmonary fibrosis [32–34]. Huang et al. demonstrated that a PAI-1 inhibitor significantly suppressed the TGF-β-induced expression of α-SMA and fibronectin in CCL-210 human lung fibroblasts [22]. Consistent with this report, we also found that SK-216 limited TGF-β-induced overexpression of α-SMA in MRC-5 cells and human primary lung fibroblasts. These results confirm PAI-1 inhibition has the potential to block TGF-β-induced differentiation of fibroblasts to myofibroblasts characterized by α-SMA expression. However, unlike the results of Huang’s report [22], PAI-1 inhibition by SK-216 did not affect the expression of fibronectin and type I collagen in MRC-5 cells or primary lung fibroblasts. Although we do not have a convincing explanation for this discrepancy, SK-216 significantly influenced α-SMA expression, but its effect on ECM production was negligible during fibroblast to myofibroblast differentiation. These observations suggest that PAI-1 is involved in TGF-β-induced differentiation into myofibroblasts, defined by α-SMA as a downstream effector.

In the present study, we have demonstrated that TGF-β-induced EMT stimulated the production of both PAI-1 and endogenous TGF-β in A549 cells and the blockade of EMT by SK-216 negated these effects of exogenous TGF-β. These results suggest that the induction of EMT by TGF-β stimulated the expression of PAI-1 and TGF-β in A549 cells and again PAI-1 acts as a downstream effector of TGF-β. In mesangial cells, PAI-1 is reported to activate the TGF-β1 gene promoter [35] and enhance the expression of TGF-β [27], suggesting that a positive feedback loop exists between PAI-1 and TGF-β. These observations imply that PAI-1 released by A549 cells treated with TGF-β enhanced the production of TGF-β, and SK-216 was effective at disrupting this positive feedback loop in the present study.

In our study, oral administration of SK-216 through drinking water was shown to limit the development of BLM-induced pulmonary fibrosis in mice. We also found that administration of SK-216 reduced active PAI-1 levels in BALF and PAI-1 mRNA expression in BLM-injured lung tissues. These results indicate that SK-216 counteracted PAI-1 activity and also reduced PAI-1 production in the lung tissue. Furthermore, administration of SK-216 reduced α-SMA expression in the BLM-injured lung at both protein and mRNA levels. This result is consistent with *in vitro* findings. These data suggest that SK-216 can exert an antifibrotic effect even *in vivo* and a therapeutic strategy targeting PAI-1 might become an option for treatment of pulmonary fibrosis. Measurement of TGF-β levels in BAL fluids on day 11 after BLM treatment (= 2 days after the start of SK-216 administration) showed that SK-216 administration did not affect TGF-β levels in BAL fluids. This result is not consistent with *in vitro* findings that SK-216 inhibited the production of endogenous TGF-β in A549 cells upon TGF-β-induced EMT. Based on this discrepancy between the *in vivo* and *in vitro* results, we can speculate that TGF-β production in bleomycin-treated mice is determined at a time point prior to the start of SK-216 administration, and/or SK-216 does not suppress TGF-β production by cells other than lung epithelial cells. Furthermore, we can speculate that the antifibrotic action of SK-216 is mediated by the inhibition of the pro-fibrotic activity of TGF-β, but not by the reduction of TGF-β production *in vivo*. We believe that the antifibrotic effect of SK-216 *in vivo* is also mediated by its inhibitory effects on TGF-β-induced EMT and differentiation of fibroblasts to myofibroblasts, but this possibility remains to be tested. Further investigations are necessary to understand mechanisms involved in *in vivo* antifibrotic effects of PAI-1 inhibitors.

In conclusion, we demonstrated that treatment of cells with a PAI-1 inhibitor, SK-216, blocked the occurrence of TGF-β-dependent EMT and differentiation of fibroblasts to myofibroblasts, suggesting that PAI-1 is a potent downstream effector of TGF-β in fibrotic processes. In addition, oral administration of SK-216 was shown to limit the development of BLM-induced pulmonary fibrosis in mice, suggesting that inhibition of PAI-1 *in vivo* exerts an antifibrotic effect. These results imply that targeting PAI-1 as a downstream effector of TGF-β can become a promising therapeutic strategy for pulmonary fibrosis.
